# Supplementary material for: Effects of Polyvinyl Chloride (PVC) Microplastic Particles on Gut Microbiota Composition and Health Status in Rabbit Livestock
Source: Int J Mol Sci. 2024 Nov 25;25(23):12646. doi: 10.3390/ijms252312646 (PMC11641588; doi:10.3390/ijms252312646)
Supplement: Supplementary file 1 [file ijms-25-12646-s001.zip › Papp et al_supplementary figures/Suppl Fig S1_organs weight values Papp et al.pdf]

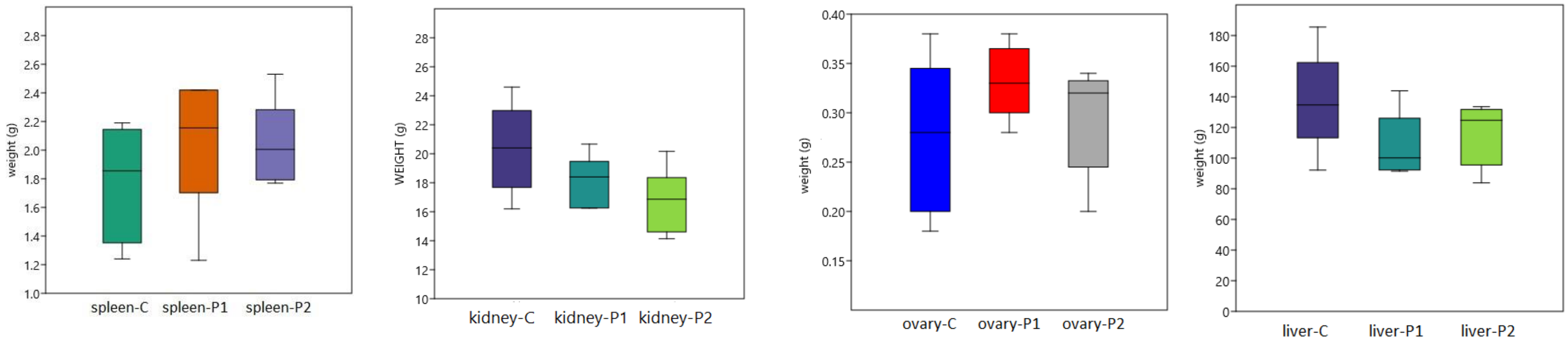

Supplementary Figure S1 Boxplots showing organs weight values in the three experimental groups: control (C), low dose (P1) and high dose (P2).
