# Supplementary material for: Effects of Polyvinyl Chloride (PVC) Microplastic Particles on Gut Microbiota Composition and Health Status in Rabbit Livestock
Source: Int J Mol Sci. 2024 Nov 25;25(23):12646. doi: 10.3390/ijms252312646 (PMC11641588; doi:10.3390/ijms252312646)
Supplement: Supplementary file 1 [file ijms-25-12646-s001.zip › Papp et al_supplementary figures/Suppl Fig S2_E2 levels Papp et al.pdf]

Supplementary Figure S2. 17β-estradiol serum levels in the different experimental groups over the weeks

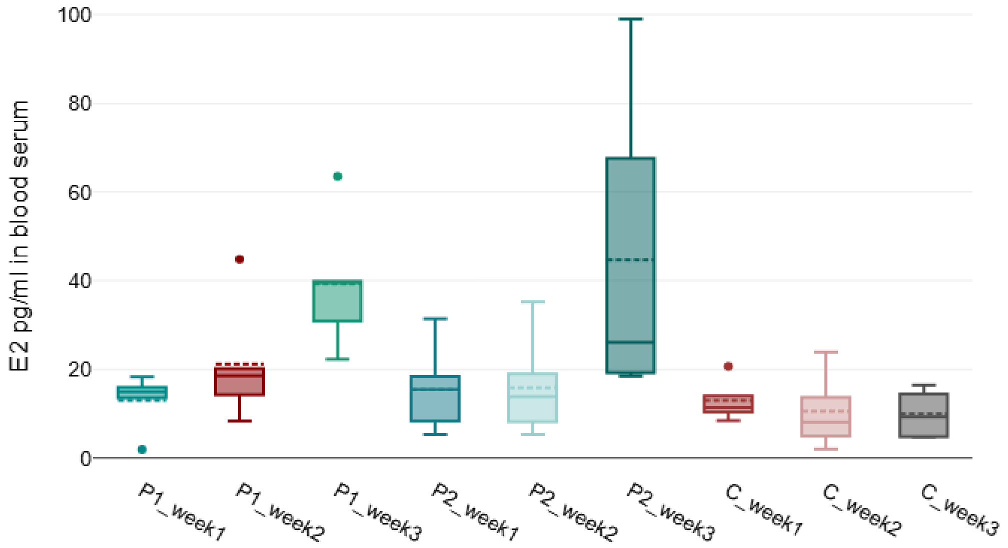


Supplementary Figure S2. The Kruskal-Wallis test was employed (Chi2-value = 19.91, df = 8.0; p = 0.011). The Dunn-Bonferroni tests revealed significant differences between the following groups: P1week1 vs P1week3 (p = 0.007), indicating that under treatment, the E2 level significantly increased in the P1 group. P1week3 vs C_week3 (p = 0.002), demonstrating that the P1 PVC treatment significantly increased E2 levels compared to the control group. P2week1 vs P2week3 (p = 0.042), suggesting that under treatment, the E2 level significantly increased in the P2 group. P2week3 vs C_week3 (p = 0.005), indicating that the P2 PVC treatment significantly increased E2 levels compared to the control group.
