# Supplementary material for: Effects of Polyvinyl Chloride (PVC) Microplastic Particles on Gut Microbiota Composition and Health Status in Rabbit Livestock
Source: Int J Mol Sci. 2024 Nov 25;25(23):12646. doi: 10.3390/ijms252312646 (PMC11641588; doi:10.3390/ijms252312646)
Supplement: Supplementary file 1 [file ijms-25-12646-s001.zip › Papp et al_supplementary figures/Suppl Fig S3_TNFalfa levels Papp et al.pdf]

Supplementary Figure S3. Trends in serum TNFα levels in the different experimental groups over the weeks

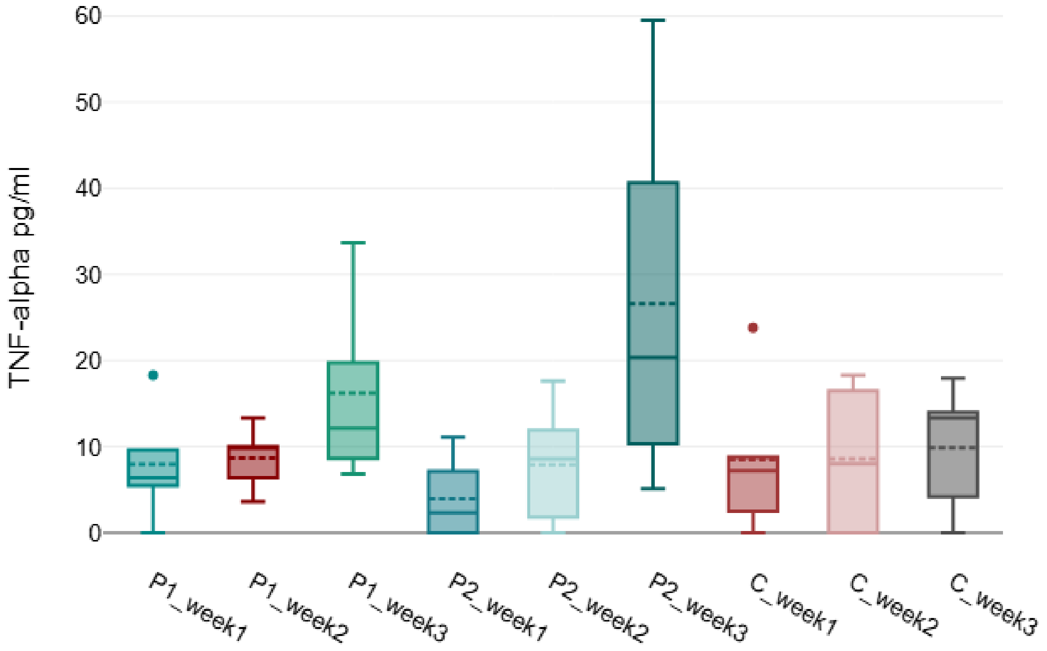

The

Supplementary Figure S3. A one-way analysis of variance revealed a significant difference between the categorical variable and the dependent variable ($F = 2.3$; $p = 0.041$). The Bonferroni post hoc test indicated that the pairwise group comparison of P2_week1 - P2_week3 demonstrated a statistically significant difference ($p = 0.024$).
