# Supplementary material for: Effects of Polyvinyl Chloride (PVC) Microplastic Particles on Gut Microbiota Composition and Health Status in Rabbit Livestock
Source: Int J Mol Sci. 2024 Nov 25;25(23):12646. doi: 10.3390/ijms252312646 (PMC11641588; doi:10.3390/ijms252312646)
Supplement: Supplementary file 1 [file ijms-25-12646-s001.zip › Papp et al_supplementary figures/Suppl Fig S4_microbiota changes Papp et al.pdf]

Supplementary Figure S4.

The phylum-level (**Figure S4A**) and family-level (**Figure S4B**) mean bacterial compositions of the rabbit ileum (I), caecum (C) and faeces (F) in the three treatment groups Control (C), P1 and P2. The most recent taxonomic nomenclature is Bacteroidota for Bacteroidetes and Pseudomonadota for Proteobacteria.

Supplementary Figure S4A Phylum-level mean bacterial compositions

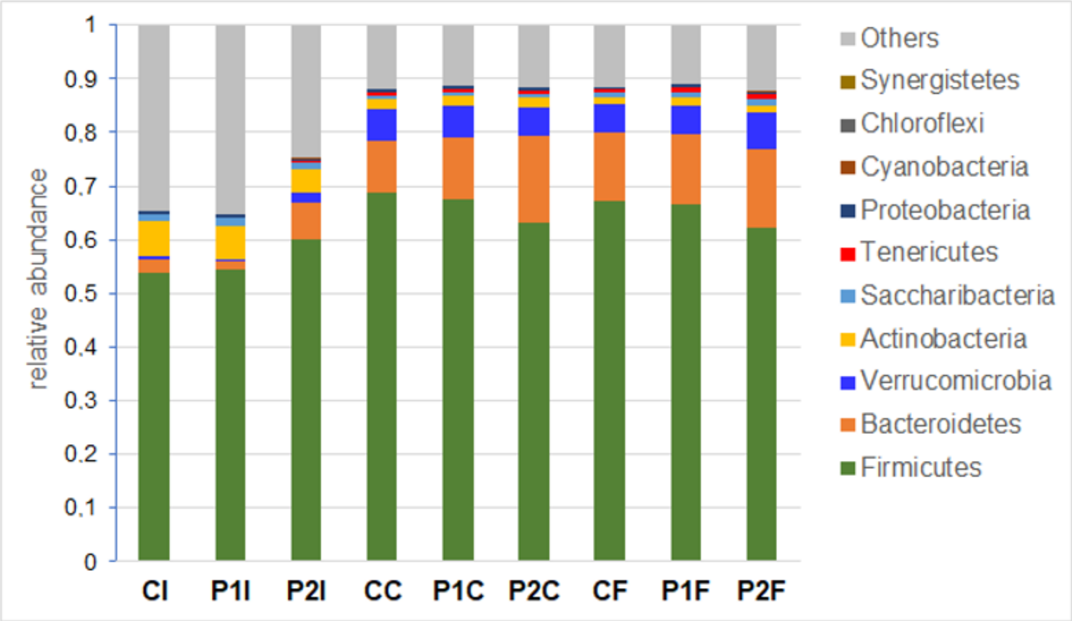

Supplementary Figure S4B Family-level mean bacterial compositions

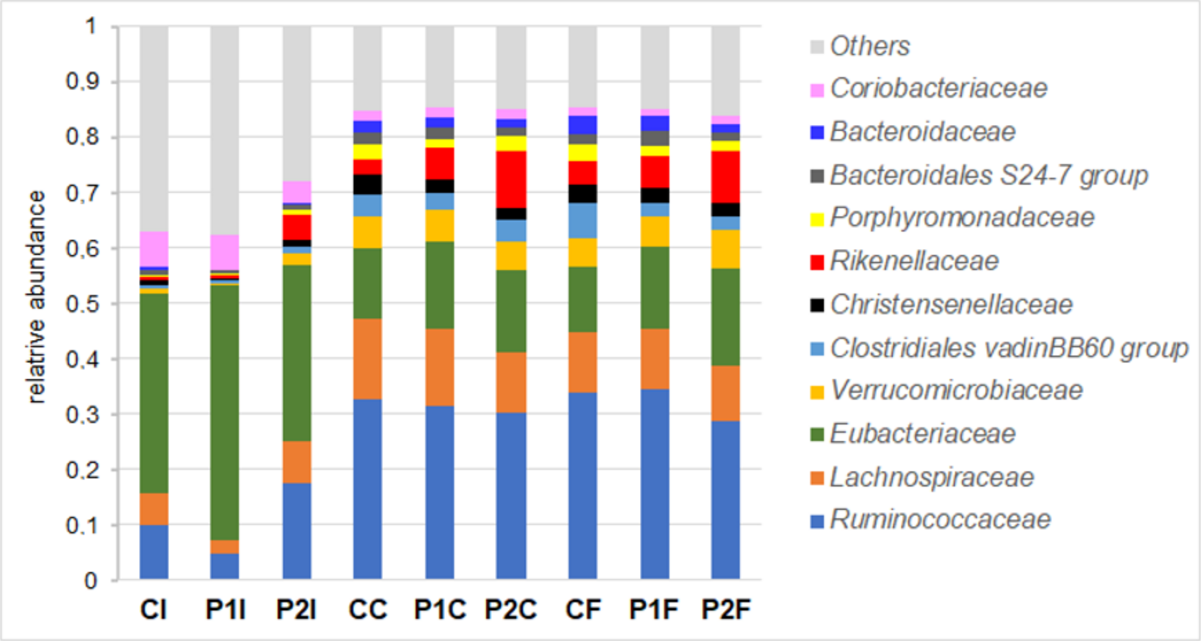

Supplementary Figure S4C Fold-changes in the mean relative abundances of the phyla Saccharibacteria, Cyanobacteria and Proteobacteria between the treatments P2 (high PVC) and the Control groups. The relative abundance change for the Proteobacteria proved significant (p=0.045) in the faeces.

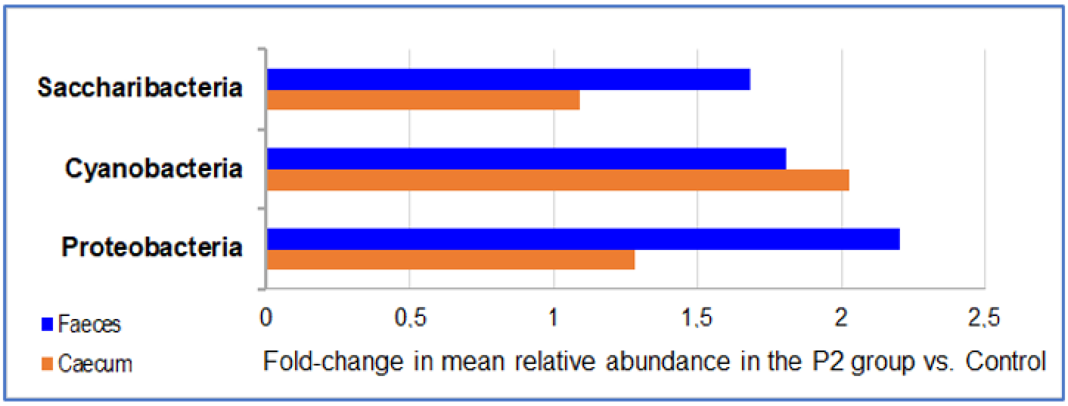

Supplementary Figure S4D. Boxplots of phylum- and ordo-level relative abundances in the rabbit faeces (F) in the three treatment groups Control, P1 and P2.

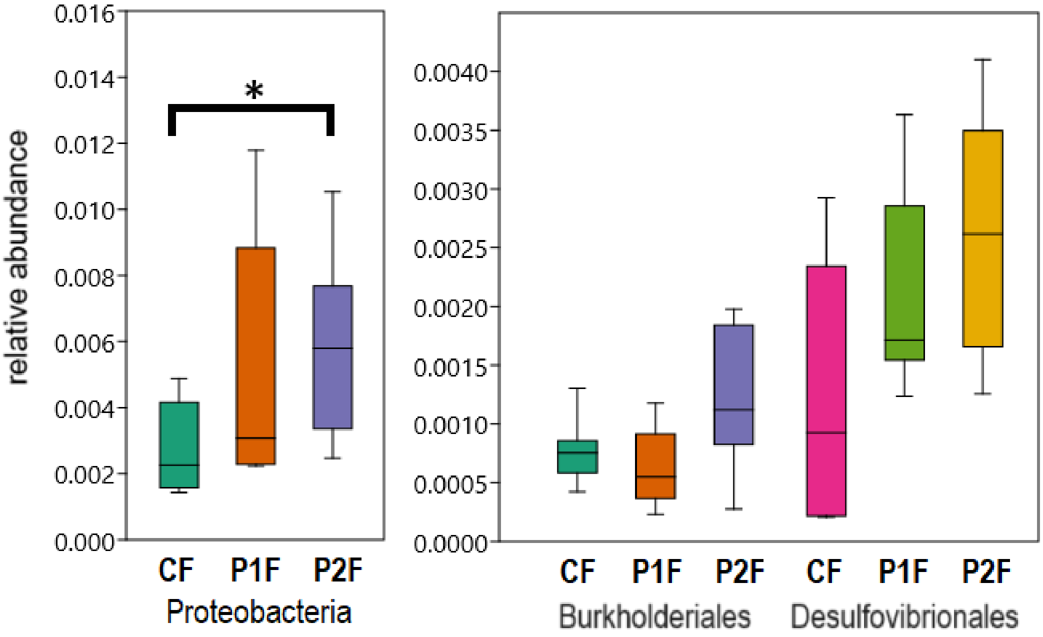

*Difference significant at the p<0.05 level
