# Supplementary material for: Effects of Polyvinyl Chloride (PVC) Microplastic Particles on Gut Microbiota Composition and Health Status in Rabbit Livestock
Source: Int J Mol Sci. 2024 Nov 25;25(23):12646. doi: 10.3390/ijms252312646 (PMC11641588; doi:10.3390/ijms252312646)
Supplement: Supplementary file 1 [file ijms-25-12646-s001.zip › Papp et al_supplementary figures/Suppl Fig S5_genus-level bar charts Papp et al.pdf]

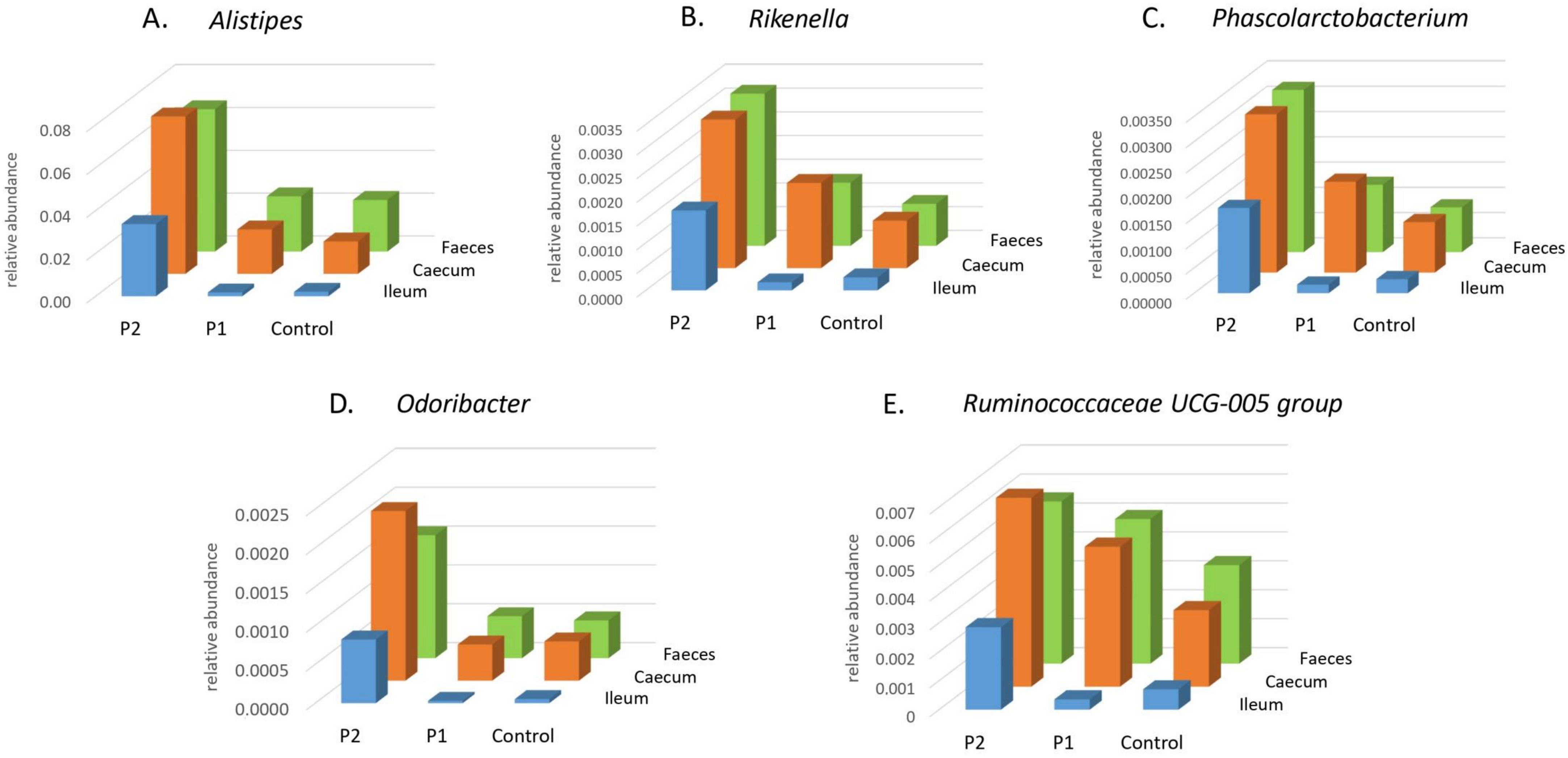

Supplementary Figure S5. Mean relative abundance values for the genus *Alistipes* (part A), *Rikenella* (part B), *Phascolarctobacterium* (part C), *Odoribacter* (part D), *Ruminococcaceae UCG-005 group* (part E) in the three treatment groups (Control, P1 and P2) for the rabbit intestinal samples ileum and caecum content and faeces. See further details in text and in Supplementary Table S6.
