# Supplementary material for: Effects of Polyvinyl Chloride (PVC) Microplastic Particles on Gut Microbiota Composition and Health Status in Rabbit Livestock
Source: Int J Mol Sci. 2024 Nov 25;25(23):12646. doi: 10.3390/ijms252312646 (PMC11641588; doi:10.3390/ijms252312646)
Supplement: Supplementary file 1 [file ijms-25-12646-s001.zip › Papp et al_supplementary tables/Table S1 PVC_mycotoxin content Papp et al.pdf]

Supplementary Table S1 PVC and mycotoxin content of feed in treatment groups

Part A The amount of PVC microplastics content of the produced feeds

**Parameter tested**	**Unit of measurement**	**C control experiment**	**P1 low dose experiment**	**P2 high dose experiment**
Amount of sample investigated	g	6.7	6.7	6.7
PP, PE, PS	particle/100g	<75	<75	<75
PMMA, PUR, PET	particle/100g	<75	<75	<75
PVC	particle/100g	<75	75	10165
PTFE	particle/100g	<75	<75	<75

The abbreviations are the followings: polyethylene (PE), polypropylene (PP), polystyrene (PS), polymethyl methacrylate (PMMA), polyurethane (PUR), polyethylene terephthalate (PET), polyvinyl chloride (PVC), polytetrafluoroethylene (PTFE),

Determination of the PVC microplastics content of the produced feeds
Analytical testing of 6.7 g of the tested feeds (C, P1 and P2) was performed by ALS Scandinavia AB (Danderyd, Sweden). The microplastic particles were identified by FTIR (Fourier transform infrared spectroscopy) and the number of micro-particles per 100 g of dry matter was calculated. The measurement of the drinking water load of the animals was also carried out in the laboratory of ALS Scandinavia AB using a similar procedure, with the difference that the number of microplastic particles was calculated per 1000 mL. The test included the detection of the following types of microplastics: polyethylene (PE), polypropylene (PP), polystyrene (PS), polymethyl methacrylate (plexi; PMMA), polyurethane (PUR), polyethylene terephthalate (PET), polyvinyl chloride (PVC), polytetrafluoroethylene (PTFE).

Part B Mycotoxin levels determined in feed (ng/g).

**Parameter tested**	**Unit of measurement**	**C control experiment**	**P1 low dose experiment**	**P2 high dose experiment**	**threshold value (**ng/g)
Total Aflatoxin (B1), B2, G1, G2	ng/g	11.87	12.05	11.64	**20**
Zearalenone	ng/g	19.0	24.9	20.2	**250**
Fumonisin B1	ng/g	<0.1	<0.1	<0.1	**5000**
Deoxynivalenol (DON)	ng/g	250.68	182.16	201.0	**900**

Determination of mycotoxin levels in feed threshold limit values are based on Commission Recommendation 2006/576/EC.
In each case, an extract was prepared from 5 g of feed. The solvent for each mycotoxin was as follows: deoxynivalenol (DON): 50 mL of distilled water for 3 minutes; Fumonisins: mixture of 30 % distilled water and 70 % methanol for 10 minutes; Zearalenone (ZEN): a mixture containing 30% distilled water and 70% methanol for 10 minutes. The extracts were filtered through a Whatman No. 1 paper filter and samples were analyzed using RIDASCREEN |DON, RIDASCREEN Fumonisin, RIDASCREEN Zearalenon ELISA assays (R-Biopharm-AG, Darmstadt, Germany) and Soft Flow Total aflatoxin ELISA kit (Pécs, Hungary). All ELISA assays were performed according to manufacturers' instructions. Reactions were measured photometrically at 450 nm with a microplate reader of ThermoLabsystem, Multiskan EX.
