# Supplementary material for: Effects of Polyvinyl Chloride (PVC) Microplastic Particles on Gut Microbiota Composition and Health Status in Rabbit Livestock
Source: Int J Mol Sci. 2024 Nov 25;25(23):12646. doi: 10.3390/ijms252312646 (PMC11641588; doi:10.3390/ijms252312646)
Supplement: Supplementary file 1 [file ijms-25-12646-s001.zip › Papp et al_supplementary tables/Table S5 blood analyses Papp et al.pdf]

Supplementary Table S5. Analyses of blood comprehensive metabolic panel

**Parameters**	**Reference values for rabbits**	**P1 low dose experimental group mean with standard deviation**	**P2 high dose experimental group mean with standard deviation**	**C control experimental group mean with standard deviation**
Albumin g/L	25-40	47.14 ± 7.56	45.82 ± 3.36	46.24 ± 2.77
Total protein (TP) g/L	50 - 75	58.78 ± 8.84	56.16 ± 3.01	58.46 ± 3.91
AST U/L	10 - 98	65 ± 14.28	76.4 ± 3.01	74.6 ± 17.24
ALT U/L	55 - 260	49.75 ± 33.15	31.6 ± 12.78	27.6 ± 12.18
ALKP U/L	100-200	76 ± 28.43	75.2 ± 60.81	103.5 ± 17.68
α-amilase U/L	200 - 500	248.5 ± 33.49	306.8 ± 23.46	288.6 ± 70.46
Lipase U/L	NA	131.4 ± 19.39	155.8 ± 13.37	147.4 ± 26.86
Glucose mmol/L	4.2 – 8.9	5.94 ± 0.18	6.04 ± 0.4	7.64 ± 1.89
Triglycerides mmol/L	1.4 – 1.76	1.22 ± 0.75	1.14 ± 0.4	1.04 ± 0.34
Total cholesterol mmol/L	0.1 – 2.00	**2.64 ± 1.77**	**2.60 ± 0.66**	**2.58 ± 0.65**
Urea mmol/L	9.1 – 25.5	7.90 ± 0.62	9.14 ± 1.61	8.04 ± 0.77
Creatinine μmol/L	53 – 124	117.4 ± 5.59	118.8 ± 7.73	**127.2 ± 8.87**
Phosphorus inorganic mmol/L	1.0 – 2.5	1.43 ± 0.29	1.77 ± 0.29	1.79 ± 0.21
Calcium, ionized mmol/L	1.71	0.78 ± 0.13	0.78 ± 0.07	0.66 ± 0.15
Potassium mmol/L	4.0 – 6.5	5.72 ± 0.65	5.45 ± 0.44	**6.64 ± 1.21**
Sodium mmol/L	130 – 155	143.5 ± 1.29	142.33 ± 0.58	144.8 ± 3.63
Chloride mmol/L	92 - 120	115.0 ± 4.08	113.0 ± 1.0	117.8 ± 6.91

The labels in the table are the followings: values underlined and in bold are below or above the reference values, respectively. NA: reference not available. The reference values are based on http://www.medirabbit.com/EN/Hematology/blood_chemistry.htm.
